# Modulating gastrointestinal microbiota to alleviate diarrhea in calves

**DOI:** 10.3389/fmicb.2023.1181545

**Published:** 2023-06-08

**Authors:** Wenjuan Du, Xianghuang Wang, Mingyang Hu, Jinxiu Hou, Yufeng Du, Wenjin Si, Linhai Yang, Le Xu, Qingbiao Xu

**Affiliations:** College of Animal Sciences and Technology, Huazhong Agricultural University, Wuhan, China

**Keywords:** calf diarrhea, gastrointestinal microbiota, gut health, rumen, probiotics

## Abstract

The calf stage is a critical period for the development of heifers. Newborn calves have low gastrointestinal barrier function and immunity before weaning, making them highly susceptible to infection by various intestinal pathogens. Diarrhea in calves poses a significant threat to the health of young ruminants and may cause serious economic losses to livestock farms. Antibiotics are commonly used to treat diarrhea and promote calf growth, leading to bacterial resistance and increasing antibiotic residues in meat. Therefore, finding new technologies to improve the diarrhea of newborn calves is a challenge for livestock production and public health. The operation of the gut microbiota in the early stages after birth is crucial for optimizing immune function and body growth. Microbiota colonization of newborn animals is crucial for healthy development. Early intervention of the calf gastrointestinal microbiota, such as oral probiotics, fecal microbiota transplantation and rumen microbiota transplantation can effectively relieve calf diarrhea. This review focuses on the role and mechanisms of oral probiotics such as *Lactobacillus*, *Bifidobacterium* and *Faecalibacterium* in relieving calf diarrhea. The aim is to develop appropriate antibiotic alternatives to improve calf health in a sustainable and responsible manner, while addressing public health issues related to the use of antibiotics in livestock.

## 1. Introduction

Neonatal calf diarrhea (NCD) is a common cause of growth disorder and death of newborn calves and leads to economic losses in the animal husbandry (Cho and Yoon, 2014). The main age affected by intestinal diseases is calves under 30 days old (Dall Agnol et al., 2021). According to the National Animal Health Monitoring Program for Dairy Products in the United States, diarrhea is responsible for 57% of weaned calf mortality, and 20% calf mortality can result in a 38% reduction in net income (Fentie et al., 2020). Neonatal diarrhea can also reduce the growth performance of animals, reduce reproductive performance and milk production in the advanced stage of lactation (Aghakeshmiri et al., 2017). In animal husbandry, antibiotics have been widely used to treat calf diarrhea and promote livestock growth. However, there is becoming increasingly clear that there are many side effects of antibiotic use, with the emergence of drug-resistant bacteria and the residue of antibiotics in meat being major concerns. More importantly, the misuse of antibiotics during the calf stage and repeated diarrhea before weaning can lead to immature rumen and intestinal flora, which can have a lasting negative impact on the digestion and absorption of the calf growing diet (Ji et al., 2018). Considering the aforementioned risks, there is an urgent need to expeditiously develop and implement innovative strategies to prevent and treat infectious diarrhea in calves. This objective seeks to minimize the requirement for antibiotic intervention and mitigate the propagation of antibiotic-resistant bacteria to both animal and human populations. Therefore, it is extremely important to reduce the use of antibiotics in calves and identify alternatives to antibiotic treatment.

**Figure 1:**
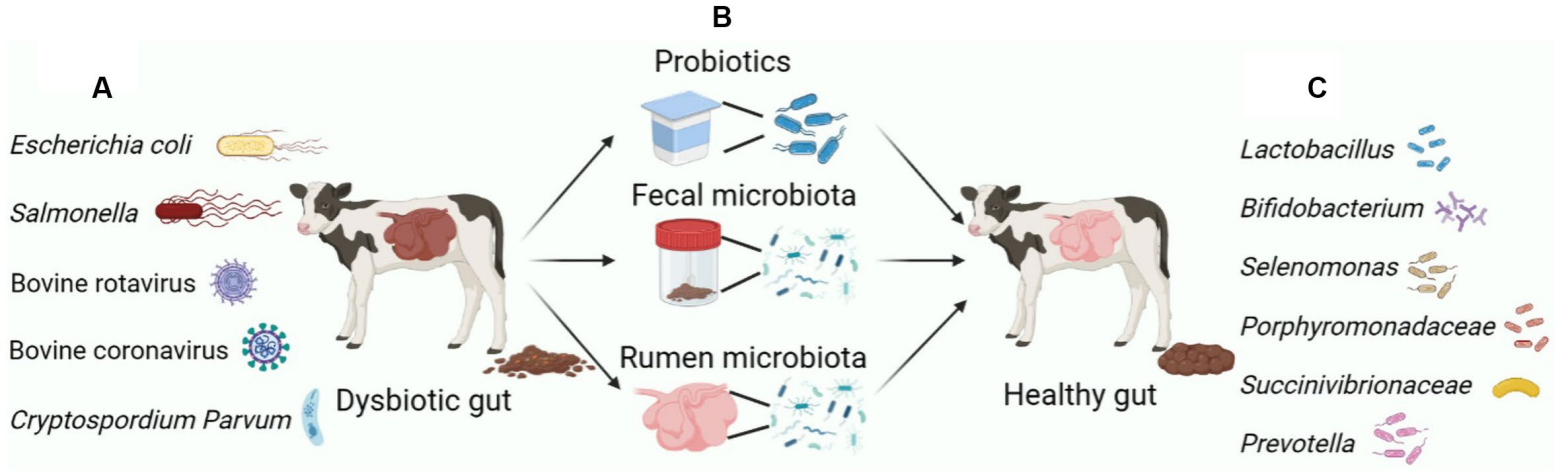
Major microbiota manipulation measures to remodel the dysbiosis of the gastrointestinal microbiota of calves. **(A)** The gastrointestinal tract of calves is susceptible to infection by pathogenic bacteria, leading to diarrhea. The main pathogenic bacteria causing gastrointestinal inflammation and diarrhea in calves include: *Escherichia coli*, *Salmonella*, BRV, BCV and *C. parvum*. **(B)** Measures to prevent and alleviate calf diarrhea through early intervention in the calf gut microbiota mainly include: Feed probiotics directly, FMT and RMT. **(C)** Probiotics mainly include *Lactobacillus*, *Bifidobacterium*, etc. The marker bacteria for normalizing the gastrointestinal microbiome of calves contain *Selenomonas*, *Porphyromonadaceae*, *Succinivibrionaceae* and *Prevotella*. Figure was created in biorender (http://biorender.io).

The early stages of animal growth have a significant impact on their health, with contact with beneficial microbiota being particularly important. The early intestinal microbiota plays a crucial role in the long-term health of the host, especially in young animals whose gut microbiota is more vulnerable to external influences. Research has shown that the early postnatal period is a critical window for manipulating the gut microbiota to optimize immunity in individual newborns (Torow and Hornef, 2017; Figure 1). The symbiotic relationship between the host and gut microbiota is vital for regulating mucosal immunity and preventing pathogen colonization. Early colonization of the gut microbiota is crucial for promoting the establishment of intestinal barrier function and the maturation of the host immune system, which are essential for maintaining overall host health (Gensollen et al., 2016). The underdeveloped immune system in newborn animals is often associated with a range of early-onset ailments, such as early diarrhea and weaning stress (Chen et al., 2018). Therefore, it is crucial to foster the maturation of the intestinal immune system during the early stages of life to enhance the growth, development, and disease resistance of newborn mammals.

Timely intervention on calves through the addition of probiotics and other methods is essential in promoting their growth and metabolism. Probiotics and prebiotics have emerged as potential alternatives to antibiotics for promoting intestinal health and reducing the incidence of calf diarrhea. A comprehensive understanding of the structure and function of gastrointestinal microbiota can help in identifying reasonable antibiotic substitutes, such as probiotics and prebiotics (Mayer et al., 2012). Evidence suggests that probiotic supplementation can decrease the incidence of diarrhea, improve average daily weight gain, and enhance feed efficiency (Timmerman et al., 2005). This review aimed to investigate early intervention of calf gastrointestinal microbiota to relieve calf diarrhea.

## 2. Current status of calf diarrhea

Calf diarrhea is a significant cause of growth disorders and mortality in newborn calves, resulting in substantial economic losses in livestock farms (Cho and Yoon, 2014). The pre-weaning phase is a critical time for calves as they are highly susceptible to infectious pathogens, which can have a detrimental impact on their intestinal health (Kim et al., 2011). Digestive disorders including diarrhea are the most common diseases of pre-weaning dairy heifers, affecting 38.5% (Urie et al., 2018). In 2018, the National Animal Health Monitoring System of the United States published research results indicating that diarrhea is the cause of 39% of calf deaths in the first 3 weeks after birth (Kim et al., 2021). The mortality rate has been recently determined to be 7.6% in Canada and 5.3% in Belgium, with 25.4% of the calves experiencing at least one disease between arrival and slaughter (Pardon et al., 2012; Winder et al., 2016). Although the mortality rate of dairy heifers in the United States decreased from 11% in 2007 to 5% in 2014, the overall morbidity rate 33.9% is still alarmingly high (Urie et al., 2018). In summary, calf diarrhea remains a practical problem that the cattle industry needs to solve urgently.

## 3. Causes of calf diarrhea

Causes of calf diarrhea are complex and multifactorial, with numerous factors contributing to its development (Cho and Yoon, 2014). Infectious agents, such as Rotavirus, Coronavirus, *Escherichia coli* and *Cryptosporidium*, are significant intestinal pathogen that cause NCD (Gulliksen et al., 2009). Calves with diarrhea are generally divided into infectious and non-infectious cases, with infectious causes being more serious and destructive to cattle husbandry. Several variables can lead to the emergence of calf diarrhea, including autoimmune disorders, malnutrition, environmental and management stress and pathogens (Cho and Yoon, 2014). During the fetal period, calves cannot obtain immuneoglobulins from the maternal circulatory system due to the placenta’s special, leading to functionally immature autoimmune systems that are easily attacked by various pathogens (Lopez and Heinrichs, 2022). An imbalance of the gastrointestinal microbiota tends to induce calf diarrhea. Currently, primary infectious agents responsible for pre-weaning diarrhea in calves include Enterotoxigenic *Escherichia coli* (ETEC), *Cryptosporidium parvum*, Rotavirus, Circovirus, and Coronavirus (Foster and Smith, 2009; Table 1). Preventing both pathogenic and non-pathogenic diarrhea during calf rearing is crucial, as illness during this stage can delay growth, impacting productivity and even resulting in death.

**Table 1:** The main pathogens that cause diarrhea in calves and their mechanism of action.

Pathogens	Period of infection	Clinical symptoms	Mechanism of action	Citations
ETEC	Calves aged 4 days after birth	Watery diarrhea, passing pale yellow, gruel-like, foul-smelling stools	ETEC proliferates in the enterocytes of the intestinal villi, causing secretory diarrhea	Foster and Smith (2009)
*Salmonella*	Calves aged 10 days to 3 months	Watery and mucoid diarrhea, passing stools that are grayish-yellow liquid mixed with mucus and blood	*Salmonella* can invade the intestinal mucosa and proliferate in the lymphoid tissue, resulting in systemic disease	Tsolis et al. (1999)
BRV	1 to 2 weeks old calves	Severe watery diarrhea with undigested curds mixed in stool	BRV replicates in the cytoplasm of intestinal villous epithelial cells, which destroys intestinal epithelial cells and secretes viral enterotoxin, resulting in diarrhea caused by indigestion and absorption	Jang et al. (2019)
BCV	1 to 2 weeks old calves	Discharge yellow watery stool with respiratory symptoms	BCV attaches to intestinal epithelial cells through prickles and hemagglutinin glycoprotein, fusing the envelope of the virus with the cell membrane or endocytosis vesicle, leading to cell death	Hodnik et al. (2020)
*C. parvum*	Calves aged 4 days and 6 weeks	Severe diarrhea, gray white or yellow feces, containing a large amount of cellulose, blood and mucus	The direct cytotoxic effect of *C. parvum* on intestinal epithelial cells and apoptosis	Buret et al. (2003)

### 3.1. Infectious factors

#### 3.1.1. ETEC

ETEC is a bacterial disease that infects newborn calves and is the leading cause of diarrhea after 4 days of birth (Ji et al., 2018). Although it rarely affects older calves or cows, severe contamination in the environment can lead to ingestion of ETEC and subsequent production of two virulence factors K99 fimbriae and heat-stable toxin (STa; Foster and Smith, 2009). The K99 antigen is expressed only at pH levels below 6.5, which STa is a primary mediator of ETEC and is secreted by many ETEC strains. Toxin production is limited when the environmental pH is below 7.0 (Saeed et al., 1986). Preventing contamination in the environment and maintaining a healthy pH level can help reduce the risk of ETEC infection in calves (Table 1). ETEC infects intestinal epithelial cells following ingestion and multiplies in the intestinal villi. The low pH of the distal small intestine creates a favorable environment for ETEC colonization, leading to villous atrophy and intrinsic damage to the lamellae (Francis et al., 1989). Upon reaching the ileum, ETEC expresses both K99 antigen and STa due to the increase in pH. Newborn calves, with higher proximal gastrointestinal tract (GIT) pH, can express the antigen more rapidly than older calves (Fossler et al., 2005). ETEC adhesion enables the bacteria to colonize and multiply in the ileum, subsequently spreading throughout the small intestine. Once established in the intestine, ETEC produces heat-stable toxins that cause secretory diarrhea (Foster and Smith, 2009).

#### 3.1.2. Salmonella

*Salmonella* is a bacterial disease that affects calves primarily from 10 days to 3 months of age and also causes diarrhea in adult cattle and heifers. The clinical manifestations of *Salmonella* are characterized by watery and mucous diarrhea with fibrin and blood (Fossler et al., 2005; Table 1). The mechanisms of *Salmonella* virulence involve the invasion of the intestinal mucosa, multiplication in lymphoid tissues, and the ability to evade the host defense system, leading to systemic disease. The pathogenesis of *Salmonella* involves the invasion of intestinal epithelial cells, the ability to survive within macrophages, and the induction of intestinal pathogenicity (Tsolis et al., 1999). *Salmonella* can colonize various regions such as M cells, intestinal cells and tonsil tissue. Once the bacteria infect lymphoid tissues such as tonsil tissue, it can quickly disseminate throughout the body by invading monocytes and phagocytes (Holt, 2000; Reis et al., 2003).

#### 3.1.3. Bovine rotavirus

Bovine rotavirus (BRV) is a significant pathogen that causes neonatal calf diarrhea, which can be severe enough to result in mortality among newborn calves (Jang et al., 2019). Typically, BRV affects calves 1–2 week old, and the short incubation period of 12–24 h cause acute diarrhea in calves. Following infection, calves can excrete significant amounts of the virus into their feces within 5 to 7 days, potentially contaminating the environment and facilitating the spread of the virus to other calves (Ammar et al., 2014). BRV infection mechanism involves replication within the cytoplasm of epithelial cells of the small intestinal villi. This result in the destruction of mature intestinal epithelial cells in the villi, activation of the enteric nervous system by the vasoactive component of the damaged cells, and the secretion of viral enterotoxins, which are the main factors contributing to rotavirus-induced dyspepsia or malabsorption leading to diarrhea (Table 1). Jang’s study demonstrated that rotavirus infection has an impact on the diversity, homogeneity, and abundance of the intestinal microbiota, as well as on the membership and structure of the microbiota (Jang et al., 2019). Moreover, a study has shown that treatment with milk replacer-based probiotics is effective in preventing clinical signs of severe diarrhea and BRV infection in calves (Kayasaki et al., 2021).

#### 3.1.4. Bovine coronavirus

Bovine coronavirus (BCV) causes calf enteritis in dairy and beef cattle. The age of the affected animals ranged from 1 day to about 3 months, with diarrhea usually occurring between 1 and 2 weeks of age and peaking between days 7 and 10 (Clark, 1993). Calves can become infected with BCV through the mouth and respiratory tract. Clinical signs usually appear 2 days after infection and last for 3 to 6 days (Hodnik et al., 2020; Table 1). Calves or calves lacking colostrum are particularly vulnerable to severe diarrhea caused by secondary coronaviruses. Coronavirus infection usually begins proximal to the small intestine and subsequently spreads throughout the small intestine (Vlasova and Saif, 2021). The virus attaches to intestinal epithelial cells via spikes and hemagglutinin glycoproteins, causing the viral envelope to fuse with cell membranes or intracellular vesicles (Schultze et al., 1991). The virus then replicates and induces cell death through release through normal secretory mechanisms, eventually leading to diarrhea (Clark, 1993).

#### 3.1.5. Cryptosporidium parvum

*C. parvum* is a common gastrointestinal pathogen affecting dairy cows and immunocompromised individuals, has become a major cause of calf diarrhea worldwide (Mosier and Oberst, 2000). Calves between 4 days and 6 weeks old often experience digestive issues due to *C. parvum* (de Graaf et al., 1999). The parasite is typically transmitted via the fecal-oral route, causing acute diarrhea (Tzipori and Ward, 2002; Table 1). The life cycle of *C. parvum* involves five stages: trophozoite, schizonts, gametophyte, zygote and oocyst, of which the oocyst is the infection stage. *C. parvum* oocysts are highly infectious and can survive in the environment for extended periods of time. Thin-walled and thick-walled oocysts are the two types of oocysts. Sporozoites escape directly into the intestinal epithelium and form self-infection in the thin-walled oocysts, whereas thick-walled oocysts (approximately 80%) sporulate in the intestinal epithelium or intestinal lumen, and the formed sporozoites are excreted with the host feces (de Graaf et al., 1999). *C. parvum* infection can cause severe villi atrophy and crypt hyperplasia in calves and other animals. This is due to the loss of intestinal epithelial cells in the villi, resulting in secondary retraction of the villi to maintain the integrity of the epithelial barrier (Argenzio et al., 1990). The increase in epithelial cell loss during *C. parvum* infection is thought to result from the direct toxic effect of organisms on intestinal epithelial cells or cell apoptosis (Buret et al., 2003).

### 3.2. Non-infectious factors

Some management factors, such as colostrum feeding, housing, calving assistance, production system, herd size, season and micro-environmental hygiene, have a significant effect on calf morbidity and mortality (Fentie et al., 2020). It is well known that an adequate supply of colostrum and passive transfer of immuneoglobulins is very important for calf health. The quantity and quality of colostrum and the timing of the first colostrum feeding play an important role (Weaver et al., 2000). Calves have low immunity and are susceptible to various infections, especially when they do not consume enough colostrum (Gitau et al., 2010). Insufficient nutrition of calves will also affect the immunity of calves, thus affecting the incidence rate and mortality (Klein-Jöbstl et al., 2014). The study found that the incidence rate of diarrhea in cattle with large-scale breeding increased because greater housing density may lead to greater disease outbreaks. It is usually recommended to place cattle houses separately because it can reduce the load of pathogens (Frank and Kaneene, 1993). The length of time that cows and calves spend in the calving area is another factor that may affect the risk of diarrhea. Studies have shown that promptly clearing the area after each calving can significantly reduce the incidence of diarrhea in farm calves (Klein-Jöbstl et al., 2014).

## 4. Calf gastrointestinal microbiota

### 4.1. Calf gut microbiota

#### 4.1.1. The importance of the gut microbiota in calves

The gut microbiota is made up of millions of genes that are essential for microbiota to survive in the gastrointestinal environment, with about 99% of them coming from bacteria (Qin et al., 2010). This fact demonstrates the importance of the gut microbiota and its indispensable role in maintaining the health and normal function of mammalian hosts (Gareau et al., 2010). The importance of gut microbiota in maintaining gastrointestinal development and function has been widely recognized, and differentiation and development of intestinal epithelial cells, mucosal layers, lymphoid structures, and immune cells are necessary for the presence of the gut microbiota (Malmuthuge and Guan, 2017). Furthermore, the establishment of the intestinal microbiota of neonatal calves is a complex process influenced by internal and external factors such as microbiota succession (Gomez et al., 2017). The symbiotic relationship between the gastrointestinal microbiota and the host is essential for maintaining mucosal immunity and defending against colonization of pathogens (Zeineldin et al., 2018). Studies have shown that the gut microbiota has a significant impact on the host immune system, and its presence early in development can have lasting effects on gut health in adults (Kerr et al., 2015). When the gut microbiota is disrupted, it leads to “ecological imbalances” that can lead to increased intestinal inflammation, impaired regulation of immune responses, reduced pathogens’ ability to compete for nutrients, and in some cases, the restoration of normal microbiota contributes to recovery from such diseases (Rolhion and Chassaing, 2016; Gomez et al., 2017). Some studies have shown that restoring a healthy microbiota community is an effective way to prevent or treat gastrointestinal diseases. Microbial culture has been used in ruminant feeding as an alternative or reduction of antibiotics for newborn calves, thereby improving cow growth performance, feed efficiency, daily gain and milk production (Krehbiel et al., 2003).

#### 4.1.2. Microbiota colonization of calves in the gut

Microbiota colonization is influenced by the bidirectional interaction between the host and microbiota, as well as external factors such as the maternal microbiota, the delivery process, diet, and antibiotic usage. Regular exposure to host-specific microbiota is essential not only for the development and maturation of the mucosal immune system but also for the absorption of nutrients and the maintenance of the animal general health (Malmuthuge et al., 2015). Oxygen in the intestine is utilized to create a strictly anaerobic environment, allowing beneficial bacteria, such as *Bifidobacterium* and *Bacteroides*, the two primary types of bacteria found in the infant gut, to thrive and have a positive effect on the mucosal immune system (Jost et al., 2012). Initial microbiota colonization of the gut of calves is typically done by *Citrobacter*, *Lactococcus*, *Leuconostoc* and *Lactobacillus* shortly after birth, and the fecal microbiota of calves at 6 and 12 h after birth is generally quite similar. However, due to increased microbiota diversity, the composition of the intestinal microbiota changes significantly from 6 to 12 h after birth (Mayer et al., 2012).

**Figure 2:**
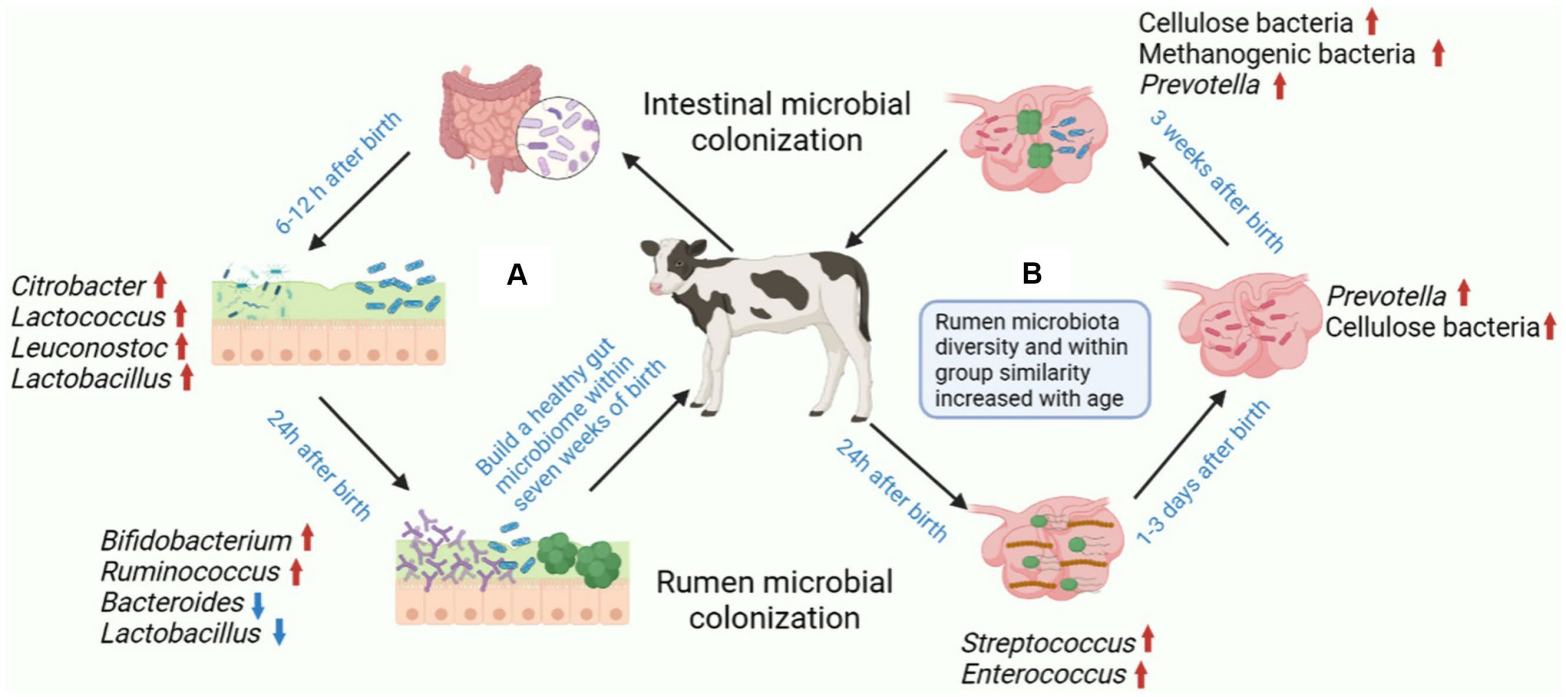
Postnatal colonization of gastrointestinal microbiota and establishment of healthy gastrointestinal microbiota in calves. **(A)** The colonization of gastrointestinal microbiota is a complex process, and the stable establishment of microorganisms is important for the host. The main colonizing bacteria in the intestines of calves 6–12 h after birth include *Citrobacter, Lactococcus, Leuconostoc* and *Lactobacillus*. The relative abundance of *Bifidobacterium* and *Ruminococcus* increased after 24 h after birth in calves, while the relative abundance of *Bacteroides* and *Lactobacillus* decreased with age. **(B)** The earliest colonizers of calf rumen were mainly *Streptococcus* and *Enterococcus*, which contributed to the transformation of the rumen into a completely anaerobic environment. The main form of rumen that can be detected in calves within 3 days of birth is *Prevotella*. Cellulose, *Methanogenic* bacteria and *Prevotella* can be detected in the rumen of calves aged 1–3 weeks. Figure was created in BioRender (http://biorender.io).

Studies have shown that the similarity in fecal microbiota composition between individuals drops sharply after 24 h, suggesting that calves quickly establish a complex microbiota soon after birth. However, the individual differences observed in calves lessen with age, indicating that a more similar microbiota community is established in the calf gut (Mayer et al., 2012). Research has demonstrated that the relative abundance of *Bifidobacterium* and *Ruminococcus* tends to increase with advancing calf age, while the relative abundance of *Bacteroides* and *Lactobacillus* decreases as calf age increases (Malmuthuge et al., 2019; Figure 2). The lower bacterial diversity in diarrheic calves compared to healthy calves implies that the gut microbiota is related to the host health. Establishing a normal gut microbiota before weaning, which generally occurs within 7 weeks of birth, is essential for calf health and growth (Oikonomou et al., 2013). Therefore, it is necessary to cultivate a healthy gut microbiota in newborn calves in order to improve gut health and overall host health.

### 4.2. Rumen microbiota in calves

#### 4.2.1. Rumen development

In ruminants, the rumen is an enlarged vestal lumen responsible for breaking down most food and is the source of major bacterial and fungal communities that ferment plant-derived lignocellulosic material and other substrates into short-chain organic acids, which are then absorbed by the host and contribute to the growth and maintenance of the animal (Flint et al., 2008). The structure and physiological traits of the rumen evolve with age, which is closely associated with the maturation of the rumen microbiota, as fermentation products participate in the formation of the villi lining the rumen wall (Beharka et al., 1998). Newborn calves have a much smaller proportion of the rumen compared to adult cows and the villi of the rumen wall, which it uses to absorb nutrients, have yet to form. During the animal lactation stage after birth, the rumen has no function and milk does not pass through it due to the reflexes of the esophageal groove. Therefore, the development of the rumen in newborn calves is necessary for optimal nutrient absorption and growth (Jami et al., 2013). As rumen development and microbiota colonization occurs, calves are physiologically transformed from pseudomonogastric animals to functional ruminants. It is notable that the development of the rumen in calves substantially affects feed intake, nutrient digestibility and overall growth. Even slight adjustments to early feeding programs and nutrition can have a major influence on the development of the rumen, leading to long-term impacts on the growth, health, and milk production of adult cattle. Therefore, the development of the rumen in newborn calves is one of the most critical aspects of calf nutrition (Diao et al., 2019).

#### 4.2.2. Colonization of the rumen microbiota

The gastrointestinal tract of young ruminants is sterile at birth, and shortly after birth, a significant number of bacteria rapidly start to colonize the forestomach. Microbiota inoculation of the rumen of newborns can occur through the vaginal canal, feces, colostrum, skin and saliva. Members of typical functional rumen groups, such as methanogens, Fibrolytic bacteria or *Proteobacteria* were detected in calf rumen less than 20 min after birth, signifying that inoculation happened even before birth (Guzman et al., 2015). *Streptococcus* and *Enterococcus* had a role at the beginning of colonizing the rumen, assisting in the formation of a completely anaerobic environment and stimulating the fast development of strict anaerobic bacteria (Jami et al., 2013). It was found that cellulolytic bacteria and other bacterial species important for rumen function can be detected as early as 1 day after birth, demonstrating the establishment of these rumen bacteria prior to the introduction of concentrated feed to young ruminants (Jami et al., 2013). Some anaerobic genera, such as *Prevotella*, that were notably more abundant were identified as permanent members of the bacterial community in the mature rumen based on samples collected from 3-day-old calves (Stevenson and Weimer, 2007; Purushe et al., 2010). Thus, calf GIT colonization is initiated early in life and may even begin during parturition as in other mammals, however, this dynamic process changes greatly during the early life history of the animal (Dominguez-Bello et al., 2010; Figure 2). The findings revealed that the rumen of three-week-old calves had a substantial level of both *Bacteroides* and *Prevotella*, indicating that fermented feed might promote the maturation of the rumen microbiota community (Malmuthuge et al., 2014).

Members of the dominant populations in the rumen, the phylum *Bacteroides* and Firmicutes were also detected as the dominant populations in the fecal community compared to the rumen bacterial community (Whitford et al., 1998). In addition, the major Rumenic fibrogenic bacteria, such as *Fibrobacterium* and *Flavibacter*, increased with calf age, which may be related to the development of the digestive tract and increased fiber digestibility after birth (Uyeno et al., 2010). At two weeks of age, the rumen microbiota in calves becomes more diverse and consists of a greater abundance of long-lived bacterial species (Li et al., 2012). Another study revealed that the diversity of rumen microbiota, as well as the within-group similarity, increased with age. This suggests a transition of the rumen microbiota from less pronounced and heterogeneous communities to more diverse and homogeneous mature bacterial communities (Jami et al., 2013). Furthermore, a recent study further supports this point, suggesting that as animals grow older, the diversity of their intestinal flora increases in terms of α diversity, but decreases in terms of β diversity (Dill-McFarland et al., 2017).

## 5. Early microbiota intervention to relieve calf diarrhea

### 5.1. Early supplement of probiotics to alleviate calf diarrhea

Probiotics are defined as living microorganisms that, if given in sufficient quantities, can provide health benefits to the host (Hill et al., 2014). Probiotics include bacteria, yeasts and fungi that have beneficial health effects on humans and animals. They have the ability to regulate the balance and activity of gastrointestinal microbiota, so they are considered beneficial to host animals and have been used as functional food (Uyeno et al., 2015). Currently, around 41% of pre-weaned heifers are using probiotics (Kim et al., 2011). Adding probiotics to the diet of young cattle can prevent imbalances in the intestinal microbiota, improve growth and prevent diarrhea, especially in stressful conditions (Frizzo et al., 2010).At present, the application of probiotics and symbiotic bacteria in young ruminants usually aims at the gastrointestinal system and minimizes the incidence of gastrointestinal diseases by stabilizing and enhancing the intestinal microbiota (Uyeno et al., 2015). Results from experiments indicate that supplementation with probiotic products can reduce the incidence of diarrhea and improve average daily gain and feed efficiency (Signorini et al., 2012).

Kim’s research has demonstrated that direct feeding of micro-organisms (including three strains of *Lactobacillus* and three *Bacillus* strains, as well as one strain of *Saccharomyces boulardii* and one strain of non-pathogenic *Escherichia coli*) as probiotics in calves after birth can significantly reduce the incidence of calf diarrhea (Kim et al., 2011; Table 2). Renaud et al. found that administering a multispecies probiotics and yeast bolus to calves at the onset of diarrhea reduced the duration of diarrhea (Renaud et al., 2019). Supplementation with a multispecies probiotic has been shown to reduce the incidence of diarrhea in neonatal calves from day 7 to 21 after birth (Wu et al., 2021). In addition, the difference of microbiota composition among young ruminants is higher than that of adult ruminants (Jami et al., 2013), and the intestinal microbiota of young ruminants may change more easily than that of adult ruminants in early life, making them more susceptible to probiotics (Abe et al., 1995). Compared with antibiotics in feed, probiotics in dairy substitutes can reduce diarrhea and serve as alternatives to antibiotics (Kim et al., 2011). Supplementing probiotics in dairy substitutes can help mitigate the adverse effects of the milk fed veal industry, especially when animals experience diarrhea (Villot et al., 2019). Thus, supplementing with probiotics offers an opportunity to improve early gut health and minimize calves’ susceptibility to intestinal infections before weaning.

**Table 2:** Alleviating calf diarrhea through early microbiological intervention.

Treatment	Microorganisms used	Effects	Citations
Direct feeding of probiotics	*Lactobacillus* *Bacillus* strains *Saccharomyces boulardii* non-pathogenic *Escherichia coli*	Reduced incidence of calf diarrhea	Kim et al. (2011)
Oral administration of microencapsulated *Lactobacillus*	*Lactobacillus* *Lactobacillus acidophilus*	Enhanced colonization of *Lactobacillus acidophilus* in the gut and reduced severity of diarrhea	Abu-Tarboush et al. (1996) and Khuntia and Chaudhary (2002)
Addition of *Bifidobacterium bifidum* to milk replacer	*Bifidobacterium bifidum*	Reduced diarrhea and improved weight gain	Sarkar and Mandal (2016)
Addition of *Saccharomyces cerevisiae* to milk substitutes	*Saccharomyces cerevisiae*	Promote the formation and transformation of rumen bacterial community and reduce the incidence of calf diarrhea	Krehbiel et al. (2003) and Villot et al. (2019)
Addition of *Bacillus subtilis* natto to calf feed	*Bacillus subtilis* natto	Improve the average daily weight gain and feed efficiency of calves, activate the immune system and enhance immunity	Sun et al. (2010)
Fecal microbiota transplantation	*Selenomonas* *Porphyromonadaceae*	Change intestinal microbiota and increase microbiota diversity and stability	Kim et al. (2021)
Rumen microbiota transplantation	*Succinivibrionaceae* *Prevotella*	Promote the establishment of early rumen microbiota	Bu et al. (2020)

### 5.2. Fecal microbiota transplantation alleviates calf diarrhea

Fecal microbiota transplantation (FMT) is a promising treatment for dysbiosis related diseases. It involves transplanting the fecal contents of a healthy donor into a diseased patient with the aim of restoring a healthy gut microbiota and reshaping it to its original state (Khoruts and Sadowsky, 2016). FMT has been demonstrated to be an effective treatment for calf diarrhea (Borody and Khoruts, 2011). The gastrointestinal tract microbiota is essential for regulating host mucosal immunity and nutrition, as well as providing resistance to pathogen colonization. Establishing a healthy microbiota is crucial for the growth and development of young animals, and when calves experience diarrhea, FMT using beneficial microbiota from healthy donors can help restore a healthy gut flora (Islam et al., 2022). Recent studies have reported that FMT is able to improve diarrhea in pre-weaned calves by modifying the intestinal microbiota and that FMT may have a potential role in improving calf growth and development (Kim et al., 2021; Table 2).

The gut microbiota has a profound impact on the development of young animals, and it has been demonstrated that changes in the gut microbiota early in life can have long-term effects on host health (Malmuthuge and Guan, 2017). Increased microbiota diversity and stability are important features of healthy calf gut microbiota development over time. Calf diarrhea can be predicted by changes in early gut microbiota to improve calf health (Ma et al., 2020). The relative abundance of *Enterobacteriaceae* bacteria in recipient calf feces at week 5 after FMT is low, suggesting that FMT may modulate gut microbiota composition early in life (Rosa et al., 2021). Studies have shown that FMT treatment in calves can decrease the occurrence of diarrhea and shift the intestinal environment of pre-weaned calves with diarrhea from an imbalanced to a symbiotic state. It was found that the intestinal microbiota of recipient calves gradually resembled that of donor calves after FMT treatment, and the relative abundance of *Porphyromonadaceae* was significantly increased, and its abundance was negatively correlated with the incidence of diarrhea, these results suggest that regulating the quantitative changes of *Porphyromonadaceae* in the calf intestine may be the key to solve diarrhea in calves (Kim et al., 2021). *Selenomonas* have also been found in successful FMT donors, suggesting that it may act as an iconic microorganism and potentially ensure donor-recipient compatibility (Islam et al., 2022). In conclusion, FMT has demonstrated effectiveness as a therapy for preventing and treating calf diarrhea through the repair of the intestinal microbiota. In addition to alleviating diarrhea symptoms, FMT may also aid in the identification of beneficial microorganisms and their functional metabolites, making it an important area for further research.

### 5.3. Early intervention of rumen microbiota promotes rumen development and relieves calf diarrhea

The early stages of a ruminant’s life offer a window of opportunity to manipulate the rumen microbiota, which could have lasting effects on the health of adult cattle (Yáñez-Ruiz et al., 2015). Previous studies have demonstrated that repeated inoculation of adult rumen microbiota in pre-weaned calves with diarrhea can significantly reduce the frequency and duration of the condition. Analysis of the rumen microbiota showed that the rumen flora of recipient calves differed from that of the donors, with only one *Succinivibrionaceae* and five other *Prevotella* were found to have a predominance of OTUs. Therefore, rumen microbiota transplantation (RMT) could be an effective approach to preventing and reducing calf diarrhea in a herd (Bu et al., 2020; Table 2). Earlier studies have shown that individual bacteria introduced via inoculation can establish and persist in the rumen of lambs fed an aseptic diet or lambs with relatively simple rumen microbiota (Fonty et al., 1983, 1997). Early dietary intervention effectively regulated the development of rumen microbiota in dairy cows, though any long-term effect on milk production was not observed after the intervention was discontinued (Saro et al., 2018; Dill-McFarland et al., 2019). Early studies used rumen fluid or contents from adult cattle to inoculate calves have also revealed that while inoculation accelerates the colonization of rumen protozoa (Pounden and Hibbs, 1950). To further explore the effects of rumen microbiota transplantation, further studies should focus on the effects of donor-recipient matching in terms of age, species and diet. In addition, future studies should also analyze the interactions between the rumen microbiota and the host, and assess the long-term effects of early dietary intervention on milk production.

## 6. Mechanism of action of probiotics

### 6.1. Probiotics competitively inhibit colonization of intestinal pathogens

**Figure 3:**
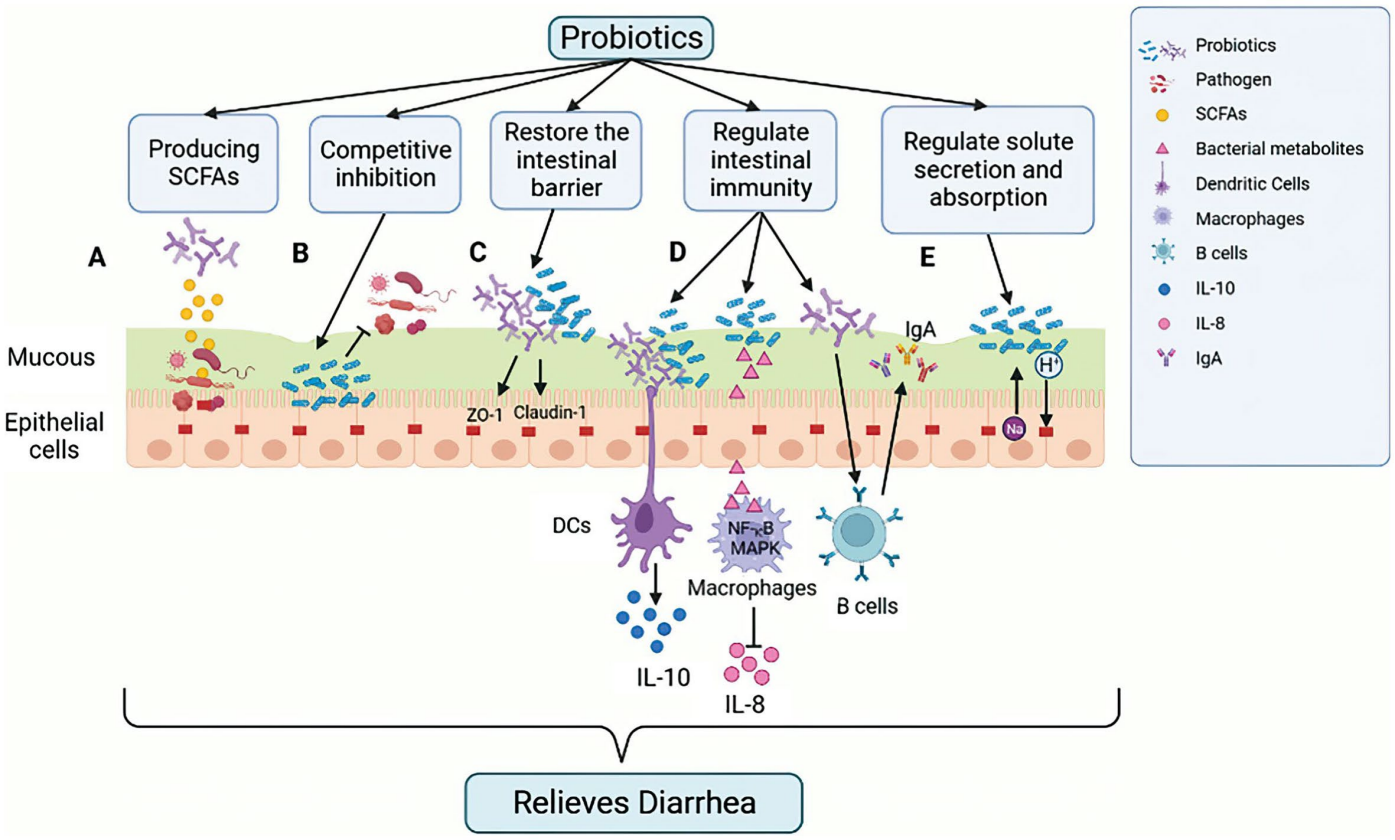
Mechanism of action of probiotics in alleviating GIT diarrhea-related inflammation in calves. Probiotics exert their effect by impacting the integrity of intestinal epithelial cells (IECs) through several mechanisms. **(A)** Production of SCFAs, lowering intestinal pH, creating a favorable environment for beneficial bacteria, and inhibiting pathogenic bacterial growth. **(B)** Competitive binding to intestinal binding sites by some probiotics, which can hinder the growth of pathogenic bacteria. **(C)** Enhancing the stability of intercellular TJ proteins, which reduces intercellular permeability of IECs to pathogens and other antigens. **(D)** Regulating the host innate and adaptive immune response, such as triggering anti-inflammatory cytokines (IL-10) secretion from DCs, inhibiting NF-κB, and reducing the levels of pro-inflammatory cytokines (IL-8) in MAPK pathway to suppress the pro-inflammatory response induced by ETEC. Probiotics can also stimulate B cells to increase the amount of IgA secreted, thus enhancing the humoral immune response and inducing an anti-inflammatory effect. **(E)** Reducing the risk of antibiotic-related diarrhea by promoting liquid absorption through the exchange of Na^+^ and H^+^ in epithelial cells. Figure was created in biorender (http://biorender.io).

In addition to the direct effects, probiotics can also modify the intestinal environment in order to gain a competitive advantage. This can be achieved through nutritional competition, by lowering the pH through the production of inhibitory compounds, or by attaching to receptor sites in the gastrointestinal tract in order to prevent pathogen binding (Neeser et al., 2000; Dicks and Botes, 2010; Figure 3B). Certain probiotic strains have adhesion proteins on their cell surface that specifically bind to carbohydrate components of the mucous layer, such as the mannose-specific adhesion mechanism of *Lactobacillus plantarum* (Pretzer et al., 2005). Probiotics can also aggregate to form a protective barrier, thus preventing the colonization of pathogenic bacteria in the intestinal epithelium (Rolfe, 2000). In addition, the growth of pathogenic bacteria can also be inhibited by the competitive rejection of intestinal binding sites by probiotics (Mekonnen et al., 2020).

Furthermore, Probiotics can promote the production of beneficial substances, such as intestinal short-chain fatty acids (SCFAs). For example, acetic acid, which is produced by probiotic *Bifidobacterium* in the gastrointestinal tract, has been shown to reduce the risk of ETEC infection (Fukuda et al., 2011; Figure 3A). Additionally, *Lactobacillus*, such as *Lactobacillus acidophilus* and *Lactobacillus plantarum*, can metabolize complex carbohydrates, such as sugars (Walter, 2008), while *Bifidobacterium* can metabolize various plant dietary fibers using various depolymerizing enzymes (Rossi et al., 2005). The use of carbohydrates other than those used by intestinal pathogenic bacteria as a source of carbohydrates allows probiotics to expand their colonization area in the GIT, thus suppressing the spread of pathogenic bacteria.

### 6.2. Probiotics restore the intestinal barrier

The intestinal barrier is a complex system consisting of the mucus layer, epithelial cell and an underlying lamina propria. The physical barrier of intestinal microbiota is formed by tight junctions (TJ) multi-protein complexes. Disruption of the tight junctions increases epithelial permeability, leading to leaky gut. The gastrointestinal barrier is a critical defense mechanism for maintaining epithelial cell integrity, preventing pathogenic infections and reducing inflammation (van Zyl et al., 2020). Probiotics have been found to upregulate the synthesis of TJ proteins, such as ZO-1 and occludin, thus protecting the integrity of the intestinal barrier (La Fata et al., 2017). For example, *Lactobacillus rhamnosus* strain GG regulates the expression and distribution of ZO-1 and claudin-1 proteins, thereby preventing damage caused by Enterohemorrhagic *Escherichia coli* O157:H7 infection (Johnson-Henry et al., 2008; Figure 3C). Studies have also shown that the probiotic *Lactobacillus plantarum* ZLP001 can increase intestinal epithelial resistance to pathogens by sustaining TJ protein abundance (Wang et al., 2018). Furthermore, *in vitro* and *in vivo* studies on different cell lines and animal models have demonstrated that probiotics such as *Lactobacillus plantarum* MB452, *Lactobacillus casei*, *Lactobacillus rhamnosus* GG and *Lactobacillus reuteri* I5007 can impact trans-epithelial electrical resistance and epithelial permeability, as well as regulate the expression and distribution of TJ proteins (Eun et al., 2008; Anderson et al., 2010; Yang et al., 2015).

### 6.3. Probiotics regulate intestinal immunity

Probiotics have a significant impact on the host innate and adaptive immunity. They can regulate various immune cells such as dendritic cells (DCs), monocytes/macrophages, T and B lymphocytes, and improve the phagocytosis of invasive intestinal pathogens (Viaşu-Bolocan et al., 2013). Probiotics also trigger the anti-inflammatory response of the innate immune system by signaling DCs to secrete anti-inflammatory cytokines, such as IL-10 (Mirpuri et al., 2012; Figure 3D). When intestinal pathogens activate NF-κB and MAPK signal pathways, it triggers the secretion of pro-inflammatory cytokines, such as IL-8, and attract inflammatory immune cells like neutrophils to the infected site, which can lead to severe inflammation and tissue damage (Llewellyn and Foey, 2017; Figure 3D). However, studies have shown that probiotic strains can inhibit the production of pro-inflammatory cytokines. For example, *Lactobacillus casei* OLL2768 can suppress ETEC-induced pro-inflammatory responses by inhibiting NF-κB and reducing the levels of pro-inflammatory cytokines in the MAPK pathway (Finamore et al., 2014). Probiotics have been reported to enhance humoral immune responses by increasing the number of IgM, IgG and IgA secretory cells and the also stimulate non-specific immune responses by activating macrophages (Isolauri et al., 2001). Probiotic supplements are used to regulate the host immune response to potentially harmful antigens. Research studies have indicated that administering *Lactobacillus* probiotics orally can raise IgA levels in children experiencing diarrhea, which can lead to a reduction in the duration of the illness (Guarino et al., 1997; Figure 3D).

### 6.4. Probiotics directly regulate solute secretion and absorption

The intestinal epithelial cells play a crucial role in regulating solute levels, and disruptions in this process can lead to watery diarrhea. Cl-secretion and Na^+^ transport, as well as uptake of Cl^−^ or HCO_3_^−^, are controlled by multiple lateral groups, apical channels and transporter proteins (Thiagarajah et al., 2015; Figure 3E). *Bacillus subtilis* CU1 has been shown to induce epithelial Na^+^/H^+^ exchange protein NHE3 expression and low levels of CFTR in mice, thereby promoting fluid absorption and reducing the risk of antibiotic-associated diarrhea (Urdaci et al., 2018). *Lactobacillus*, through its absorptive role, can also act as an antidiarrheal agent by increasing the function and expression of NHE3 (Singh et al., 2012). Probiotics, therefore, have the potential to alter intestinal electrolyte transport proteins, which are an effective mechanism to prevent antibiotic-associated diarrhea by maintaining the luminal solute concentration (Mekonnen et al., 2020).

## 7. Application of probiotics in calf diarrhea

### 7.1. Lactobacillus

*Lactobacillus* is a probiotic that inhibits the growth of pathogenic bacteria in the intestine by lowering pH and competitive attachment in the intestine (Riddell et al., 2008). Research has shown that adding *Lactobacillus* to calf diets can reduce the incidence of calf diarrhea (Abu-Tarboush et al., 1996). *Lactobacillus* also increases the total concentration of serum immunoglobulins, suggesting that it has a positive effect on calf health (Al-Saiady, 2010). Additionally, multiple *Lactobacillus* species induced greater weight gain in less healthy calves, and calves-specific probiotic treatment reduced the incidence of diarrhea and fecal coliforms counts, as reported by health scores (Timmerman et al., 2005). Supplementation of *Lactobacillus acidophilus* in 1-day-old calves has also been found to reduce the incidence and severity of diarrhea (Khuntia and Chaudhary, 2002; Table 2). Two of the most abundant *Lactobacillus* species found in healthy calves, *Lactobacillus reuteri* and *Lactobacillus johnsonii*, are being studied as potential probiotics to improve gastrointestinal health in pre-weaned calves (Fan et al., 2021).

### 7.2. Bifidobacterium

Administering *Bifidobacterium* and *Lactobacillus* to calves during the first week after birth can have significant benefits, including increased weight, improved feed conversion rates and reduced incidence of diarrhea (Abe et al., 1995). These findings suggest that these two types of bacteria are essential for maintaining intestinal balance and preventing infections in the intestines. *Bifidobacterium*, in particular, is one of the earliest and most abundant bacteria to colonize the neonatal gut and can provide various benefits to gut, metabolic and immune health. *Bifidobacterium bifidum* has been found to synthesize extracellular polysaccharides that can inhibit the growth of pathogens and protect the host epithelial cells from invasion (Sarkar and Mandal, 2016; Table 2). Recent results on FMT between young and old mice suggest that *Bifidobacterium animalis* is associated with promoting intestinal tract and parenteral health and aging in young and older mice receiving young donor microbiotas (Sarkar and Mandal, 2016). Therefore, *Bifidobacterium* may play a crucial role in preventing calf diarrhea. Studies have revealed that *Bifidobacterium pseudocatenulatum* LI09 and *Bifidobacterium catenulatum* LI10 can potentially alleviate liver injury in mice by reducing intestinal dysbiosis and preserving intestinal barrier function. By improving intestinal barrier function, the translocation of bacteria can be reduced, leading to a downregulation of an overactive immune response, ultimately preventing liver injury (Fang et al., 2017). Furthermore, research indicates that *Bifidobacterium* can significantly antagonize *Enterobacteriaceae* and *Enterococcus* by producing acetate, potentially protecting neonates from enteropathogenic infections (Nagpal et al., 2017).

### 7.3. Faecalibacterium

Research has found that *Faecalibacterium* is a microbiota biomarker present in calf feces, which has been associated to a decrease in the incidence of diarrhea and an increase in body weight during the first few weeks of life (Oikonomou et al., 2013). The administration of *Faecalibacterium prausnitzii* orally has been shown to be effective in reducing diarrhea incidence and related mortality in pre-weaning calves for up to 7 weeks after birth (Foditsch et al., 2015). *Faecalibacterium* is one of the major producers of butyrate in the large intestinal, butyrate enhances the integrity of the intestinal epithelial barrier. The relative abundance of *Faecalibacterium* has been found to be negatively correlated with the incidence of diarrhea in calves, suggesting that a high prevalence of *Faecalibacterium* early in the species may reduce susceptibility to intestinal infections (Oikonomou et al., 2013). Uyeno’s research has shown that a high abundance of *Faecalibacterium* in calves is associated with a low incidence of diarrhea in the first 4 weeks after birth. *Faecalibacterium* has an anti-inflammatory effect due to its secreted metabolites that can inhibit the activation of NF-κB and reduce the production of IL-8 (Oikonomou et al., 2013).

### 7.4. Saccharomyces cerevisiae

*Saccharomyces cerevisiae*, a type of yeast, has been found to be particularly beneficial for maintaining gut health in milk-fed calves. It can reduce the abundance of *Clostridium difficile*, the main pathogen associated with infectious diarrhea in calves, and help combat other pathogenic microorganisms (Kim et al., 2011). Research has shown that administering multiple probiotic and yeast pellets to calves during diarrhea can reduce the duration of diarrhea (Renaud et al., 2019). Supplementing calves with probiotics, including *Saccharomyces cerevisiae*, before and after weaning can also promote the formation and transition of rumen bacterial communities, facilitating the transition from liquid to dry feeds (Krehbiel et al., 2003). The fermentation products of *Saccharomyces cerevisiae* have been shown to enhance rumen morphology and exert a beneficial effect on the composition of the rumen microbiota (Xiao et al., 2016). Adding *Saccharomyces cerevisiae* to milk substitutes and concentrate has been found to reduce the incidence of diarrhea in calves and to maintain a healthy gut microbiota composition, which is important for maintaining gut health (Villot et al., 2019; Table 2). These findings suggest that *Saccharomyces cerevisiae* can help calves overcome the challenging times of early life in the milk-fed calf industry.

### 7.5. Bacillus subtilis

*Bacillus subtilis* has been found to have multiple benefits for calves, including altering the rumen microbiota, improving digestion at weaning and decreasing the severity of diarrhea (Ushakova et al., 2013). Kim’s research also supports the use of probiotics, including three strains of *Lactobacillus*, three strains of *Bacillus*, one strain of *Saccharomyces boulardii* and one strain of non-pathogenic *Escherichia coli*, for reducing the incidence of calf diarrhea when directly fed postnatally (Kim et al., 2011). Feeding the calves directly with *Bacillus subtilis* before weaning can improve the growth performance of calves, including average daily gain and feed efficiency. In addition, the *Bacillus subtilis* natto can also induce the secretion of serum IgG and Th1 cytokines in calves, including IFN-γ, This helps to activate the immune system and enhance immunity (Sun et al., 2010; Table 2). These findings suggest that *Bacillus subtilis* can be a useful probiotic for promoting calf health and reducing the risk of diarrhea.

## 8. Discussion and prospect

Controlling the intestinal microbiota through direct feeding of microorganisms, probiotics has been extensively researched in animal husbandry to modify rumen fermentation and prevent pathogen colonization. This approach has led to improving production and better health outcomes in animals. Among these interventions, oral probiotics have been found to be an effective means of alleviating diarrhea in calves, with numerous studies demonstrating their ability to restore dysbiosis of the intestinal microbiota. Therefore, it is important to explore the potential of various beneficial bacteria in the early development of the intestinal tract to enhance the host health. By establishing a microbiota community dominated by beneficial bacteria, the colonization of intestinal pathogens after birth may be impeded, thereby safeguarding the immune system of infant ruminants from intestinal infections.

## 9. Conclusion

Diarrhea is the leading cause of death in calves during the first month of life. The gut microbiota of newborn calves changes in the early postnatal period, and homeostasis of the gut microbiota ecosystem is critical for maintaining gastrointestinal function in calves until early weaning. Therefore, it is very important to establish a healthy calf gastrointestinal microbiota using early microbiota intervention methods. Supplementation with probiotics is an effective way to improve early gut health and minimize the susceptibility of calves to gut infections before weaning, thereby reducing diarrhea rates in calves and potentially affecting calf growth and development long-term effects.

## Author contributions

QX and WD conceptualized the review. WD wrote this manuscript. MH, JH, YD, WS, LY, XW, LX, and QX revised the main manuscript. All authors contributed to the article and approved the submitted version.

## Funding

This work was funded by the grants from the National Key R&D Program of China (no. 2022YFD1301004 and 2022YFD1300705), and Key Laboratory of Molecular Animal Nutrition of Zhejiang University (KLMAN202101 and KLMAN202205).

## Conflict of interest

The authors declare that the research was conducted in the absence of any commercial or financial relationships that could be construed as a potential conflict of interest.

## Publisher’s note

All claims expressed in this article are solely those of the authors and do not necessarily represent those of their affiliated organizations, or those of the publisher, the editors and the reviewers. Any product that may be evaluated in this article, or claim that may be made by its manufacturer, is not guaranteed or endorsed by the publisher.
